# Intolerance of uncertainty and repetitive negative thinking: transdiagnostic moderators of perfectionism in eating disorders

**DOI:** 10.1186/s40337-024-01138-1

**Published:** 2024-11-04

**Authors:** Craig Hyde-Smith, Holly Carey, Trevor Steward

**Affiliations:** 1 Melbourne School of Psychological Sciences, Faculty of Medicine, Dentistry and Health Sciences, University of Melbourne, Parkville, VIC Australia; 2 Department of Psychiatry, Faculty of Medicine, Dentistry and Health Sciences, University of Melbourne, Parkville, VIC Australia

**Keywords:** Eating disorders, Perfectionism, Intolerance of uncertainty, Repetitive negative thinking

## Abstract

Perfectionism has been identified as a vulnerability and maintenance factor across eating disorders (EDs). Additionally, research has found that intolerance of uncertainty (IU) and repetitive negative thinking (RNT) moderate the relationship between perfectionism and psychopathology. Despite this evidence, few studies have examined if these factors specifically moderate ED severity. This study aimed to fill this gap by investigating the moderating effect of IU and RNT on the relationship between perfectionism and ED symptoms, as well as the predictive power of these factors on identifying participants with clinically significant ED symptoms. 331 treatment-seeking adults from a university-affiliated community clinic and 264 undergraduate students completed measures to assess ED symptoms, RNT, perfectionism and IU. 147 participants (24.7% of the total sample) had clinically significant levels of ED symptoms as determined by the Eating Disorder Questionnaire Short-Form (EDE-QS). Multiple linear regressions found significant associations between ED symptoms and both the IU*perfectionism interaction (*p* < .001) and the RNT*perfectionism interaction (*p* < .001). A binary logistic regression identified that both the IU*perfectionism interaction (*p =* .03) and the RNT*perfectionism interaction (*p =* .001) predicted clinically significant EDE-QS scores. The findings indicate that both IU and RNT moderate the relationship between perfectionism and ED symptoms and build on previous literature recognising transdiagnostic contributors to ED symptomatology.

**Supplementary Information:**

The online version contains supplementary material available at 10.1186/s40337-024-01138-1.

## Abstract (plain language summary)

Perfectionism, intolerance of uncertainty and repetitive negative thinking have been shown to maintain symptoms across multiple eating disorders. This is the first study to investigate the influence of intolerance of uncertainty and repetitive negative thinking on the relationship between perfectionism and eating disorder symptoms in a treatment-seeking sample. We found that both intolerance of uncertainty and repetitive negative thinking impact the relationship between perfectionism and eating disorder symptomatology. By better understanding the role of these constructs, we can develop more effective and personalized treatments.

**Supplementary Information:**

The online version contains supplementary material available at 10.1186/s40337-024-01138-1.

## Background

The transdiagnostic theory of eating disorders (ED) aims to identify shared psychopathological processes that underly and maintain different ED diagnoses [1, 2]. A leading transdiagnostic theory proposed by Fairburn et al. [2] suggests that EDs are maintained by a core process involving a dysfunctional evaluation of self-worth according to eating, shape and/or weight. This theory also suggests that, in some individuals, perfectionism, core low self-esteem, mood intolerance and interpersonal difficulties, are key maintaining processes. A body of research has since supported the validity of this theory [3, 4] and enhancing current best-practice treatment to target these processes has delivered promising results [1]. However, in order to ensure its theoretical validity and to fortify its clinical utility, it is imperative to attain a comprehensive grasp of the pertinent processes at play.

### Perfectionism and eating disorders

Perfectionism is defined as the setting of impossibly high standards, the rigid pursuit of those standards, fears over making mistakes and an over-evaluation of self-worth based on the ability to meet those standards [5, 6]. There are several lines of evidence suggesting perfectionism is both a risk and maintenance factor for EDs. Firstly, individuals with EDs tend to have higher levels of perfectionism compared to healthy controls [5]. Secondly, studies suggest that premorbid perfectionism is a risk factor for the development of EDs [7, 8]. Lastly, enhancing best-practice treatments to additionally target perfectionism has shown improved clinical outcomes [1, 2, 9]. Perfectionism levels appear to differ minimally between patients with anorexia nervosa and bulimia nervosa, supporting the notion of it being a transdiagnostic eating disorder factor [5, 10]. Furthermore, high levels of perfectionism appear to be a negative predictor of treatment outcomes, especially in patients with anorexia nervosa [11, 12].

Research suggests that may be several factors moderating the relationship between perfectionism and psychological distress [13, 14]. However, most of these studies have featured populations with anxiety and depression, and few studies have investigated the moderators of perfectionism as it relates to ED symptoms [5]. As such, identifying the factors that moderate this relationship constitutes an important step in improving the field’s understanding of transdiagnostic ED factors.

### Intolerance of uncertainty and perfectionism

One construct that has been frequently found to moderate the relationship between perfectionism and psychological distress is intolerance of uncertainty (IU) [15]. People with high levels of IU tend to perceive uncertainty as negative and threatening, potentially leading to avoidance, doubt, and maladaptive coping strategies [16, 17] and increased IU is common across a range of psychiatric diagnoses [18].

A systematic review of 21 studies and a meta-analysis of five studies found that individuals with EDs had significantly higher levels of IU compared to healthy controls [19]. It may be the case that individuals with elevated levels of both perfectionism and IU find uncertainty threatening for fear of not living up to high expectations or of not being able to adapt to the unknown. As a consequence, they may then engage with ED behaviours to cope with this distress [20]. Despite this, few studies have looked at the potential moderating effect of IU on the relationship between perfectionism and ED symptoms.

Brosof et al. [21] investigated whether the interaction of IU and perfectionism accounted for the presence of ED symptoms and found that IU significantly moderated the relationship between personal standards perfectionism and ED symptoms both cross-sectionally and prospectively. Specifically, high personal standards perfectionism was associated with ED symptoms only when accompanied with high IU scores. The authors argue that, for these individuals, uncertainty may endanger their capacity to uphold rigorous standards and could potentially lead to the adoption of disordered eating behaviours as a coping mechanism. However, it’s worth noting that this study used a community sample, and it is unclear if the same moderating effect applies to treatment-seeking clinical populations. Furthermore, the study investigated the specific moderating effect of high standards perfectionism and not perfectionism more broadly.

### Repetitive negative thinking and perfectionism in eating disorders

Repetitive negative thinking (RNT) is a style of thinking about one’s past, present and/or future in a way that is predominately negative, repetitive, intrusive, and difficult to disengage from [22]. Several studies have found RNT to be an important vulnerability and maintenance process involved in core ED symptoms [23] and one meta-analysis of 43 studies found a significant association between RNT and clinical EDs [24]. Additionally, the authors found that RNT was associated with ED symptoms in both clinical and non-clinical samples.

Interestingly, Flett et al. [25] found that in a University sample, the relationship between perfectionism and psychological distress became non-significant when controlling for RNT, and another study found RNT to be a partial moderator between perfectionism and psychological distress [22]. RNT has been suggested as a potential moderator for perfectionism in that it intensifies perfectionism’s impact by amplifying concerns about meeting elevated standards, the likelihood of failure, and persistent rumination on past mistakes [22, 26]. Despite these findings, there has been limited research on the impact of RNT on ED symptoms, with one study by Rivière et al. [27] indicating that rumination moderated the relationship between ED symptoms and perfectionism. However, its reliance on a community sample raises uncertainty about the generalisation of this effect to clinical presentations.

### The current study

This study aims to investigate the interaction effect of IU and RNT on the relationship between perfectionism and ED symptoms in an adult treatment-seeking sample and an undergraduate student sample. We hypothesised that the interaction between IU and perfectionism would function as a partial moderator of the relationship between perfectionism and ED symptoms. Similarly, we hypothesised that the interaction between RNT and perfectionism would function as a partial moderator of the relationship between perfectionism and ED symptoms.

## Method

### Participants

Clinical participants (*n* = 492) were treatment-seeking patients at the University of Melbourne Psychology Clinic (UMPC) who were recruited between 2018 and 2022. The UMPC provides outpatient psychological treatment to the local community, university students and staff. Upon intake, participants completed the questionnaires listed below. In addition, the majority of clinical participants (*n =* 390) completed a structured clinical interview (Structured Clinical Interview for DSM-5 Disorders or Diagnostic Interview for Anxiety, Mood, and OCD and Related Neuropsychiatric Disorders) [28, 29]. Of the participants included in the analysis who completed a clinical interview (*n* = 329), 32 (9.7%) met criteria for possible binge eating disorder, 4 (1.2%) for anorexia nervosa and 7 (2.1%) for bulimia nervosa. The most common diagnoses were generalised anxiety disorder (*n* = 155), major depressive disorder (*n* = 145) and social anxiety disorder (*n* = 98). A correlation analysis was run to understand the relationship between scores on the Eating Disorder Questionnaire Short Form (EDE-QS) and number of comorbidities. The results suggested a weak positive linear relationship between total number of diagnoses and total scores on the EDE-QS. Further information about the diagnostic interview results is provided in the supplementary materials (S1).

264 participants were undergraduate students from the University of Melbourne who received course credit for completing the same questionnaires as the clinical sample described above. These non-treatment-seeking participants did not complete a structured clinical interview.

Data from 161 clinical participants was excluded because of incomplete and/or invalid responses on the EDE-QS, FMPS, IUS-SF or RNTQ, leaving a total sample of 595 participants (331 clinical participants and 264 undergraduate student participants) for our analyses (S2). Total sample and sub-sample characteristics are summarised in Table 1. The age of the undergraduate sample was significantly younger than the clinical sample (*p* < .*05*, d = 1.18, indicating a large effect). As such, age was included as a covariate in our regression analyses and as a predictor variable in the logistic regression analysis.

The present study was carried out in accordance with the latest version of the Declaration of Helsinki. The University of Melbourne Human Research Ethics Committee approved this study (HREC: 12813) and signed informed consent was obtained from all participants.

**Table 1:** Demographics and behavioural information for total, clinical and undergraduate sample

	Total sample *(n* = *595)*	Clinical sample *(n* = *331)*	Undergraduate student sample *(n* = *264)*
Age	22.81 ± 6.59	25.74 ± 7.35	19.15 ± 2.81
Sex			
Female Male Not identifying as male or female	444 (75%) 146 (24%) 5 (1%)	236 (71%) 90 (27%) 5 (2%)	208 (79%) 56 (21%) 0 (0%)
Measures			
EDE-QS FMPS IUS-SF RNTQ	9.24 ± 8.07 73.75 ± 16.90 34.61 ± 10.76 84.22 ± 27.70	8.34 ± 7.26 74.34 ± 15.94 35.42 ± 10.57 93.04 ± 23.07	10.35 ± 8.86 73.02 ± 18.02 33.61 ± 10.92 73.26 ± 29.06

*Note* EDE-QS (Eating Disorder Questionnaire Short Form), FMPS (Frost Multidimensional Perfectionism Scale), RNTQ (Repetitive Negative Thoughts Questionnaire), IUS-SF (Intolerance of Uncertainty Scale), mean ± standard deviation

### Measures

#### Eating disorder questionnaire short-form (EDE-QS)

The EDE-QS [30] is a 12-item self-report measure that has been adapted from the 28-item Eating Disorder Examination Questionnaire [31]. The EDE-QS assess an individual’s level of eating disorder symptomology. Participants are asked to reflect on how frequently they have engaged in specific eating related cognitions, emotions, or behaviours over the last seven days (i.e. ‘*On how many of the past seven days have you had a definite fear that you might gain weight?’)*. Internal consistency in our sample was excellent with a Cronbach’s α of 0.91. McDonald’s omega for the total score of the EDE-QS was found to be 0.92, further indicating high reliability. According to Prnjak et al. [32] scores of 15 or above on the EDE-QS indicate clinically significant eating disorder symptoms. 147 participants (24.7% of the total sample) had EDE-QS scores of 15 or above. 24 of the participants with an ED diagnosis (16 with possible binge eating disorder, 3 with anorexia nervosa and 5 with bulimia nervosa) had an EDE-QS score of 15 or above.

#### Frost multidimensional perfectionism scale (FMPS)

The FMPS is a validated 35-item multi-dimensional self-report measure used to assess perfectionism [33, 34]. Traditionally, the FMPS has been a 35-item measure with 6-subscales: concern over mistakes, personal standards, parental expectations, parental criticism, doubts about actions and organisation. However, Stallman et al. [35] found that a 29-item 5-factor model which combines parental expectations and criticism was more stable. The 29-item version was used for this study and our sample had excellent internal consistency with a Cronbach’s α of 0.92. The McDonald’s omega for the total score was 0.95, reflecting excellent reliability. Total scores are calculated by combining all the subscale scores except organisation [33]. Higher scores indicate higher levels of perfectionism.

#### Intolerance of uncertainty scale short-form (IUS-SF)

The IUS-SF [36] is a 12-item self-report measure that has been adapted from the 28-item Intolerance of Uncertainty scale [37]. The IUS-SF measures an individual’s perceptions of, and ability to, tolerate uncertainty. Participants were asked to indicate the extent to which a range of statements (i.e. *‘unforeseen events upset me greatly’*) are characteristic of them. The internal consistency of the IUS-SF for our sample was excellent (α = 0.93). The reliability was further assessed using McDonald’s omega. The results indicated strong internal consistency, with an omega total of 0.94.

#### Repetitive negative thoughts questionnaire (RNTQ)

RNTQ [38, 39] is a 22-item self-report questionnaire used to measure an individual’s level of repetitive negative thinking. Participants were presented with a range of statements (i.e. ‘*I am often stuck in my head*,* unable to function’)* and asked to rate how typical they are for them. The measure contains 4 subscales: worry, brooding/rumination, thought interference and pessimistic fixation. The measure demonstrated excellent internal consistency (α = 0.98) and good 3-month test-retest reliability (*r* = .76). Internal consistency for the current sample was excellent with a Cronbach’s α of 0.97 and a McDonalds omega total of 0.95.

### Procedure and analysis

All data analysis was conducted using RStudio. A summary of the correlations between the variables and the sub-samples is provided in the supplementary materials (see S3). Given the significant age difference between the samples (Table 1), regression analyses on standardised estimated were run to ensure that these factors were not significant covariates (S4). All variables failed to pass Shapiro-Wilks normality tests (S5). When assessing a quantile-quantile plot, all measures expect for EDE-QS were normally distributed. Additionally, all models passed linearity assumptions (S6). Collinearity was acceptable between the measures in our models (S7). Assumption check results are provided in the supplementary information.

A multiple linear regression analysis was used to test for our first hypotheses. Akaike (AIC) and Bayesian Information Criterion (BIC) were used to select the best fitting model for both hypotheses (see S8). Based on the secondary aim and assumption results (particularly due to the compromised normality test for EDE-QS scores) a binary logistic regression was chosen for hypotheses 3 and 4. The binary dependent variable was the presence or absence of a clinically significant EDE-QS score (≥ 15) [32]. Odds ratios with 95% confidence intervals were calculated to understand the relative association between each predictor variable and the outcome variable. Odds ratios were run to better understand the relative association between the different variables. Wald Chi-Square tests were used to understand whether or not each variable significantly improved the model’s predictive ability. The two models most relevant to our hypotheses and included in the manuscript had EDE-QS scores as the outcome variable and age, FMPS scores and the relevant interaction as predictor variables. We also ran three other models with different combinations of the predictor variables to aid in further understanding the data, which have all been included in the supplementary material (S9).

## Results

### Interaction between perfectionism, IU and ED symptoms

**Fig. 1:**
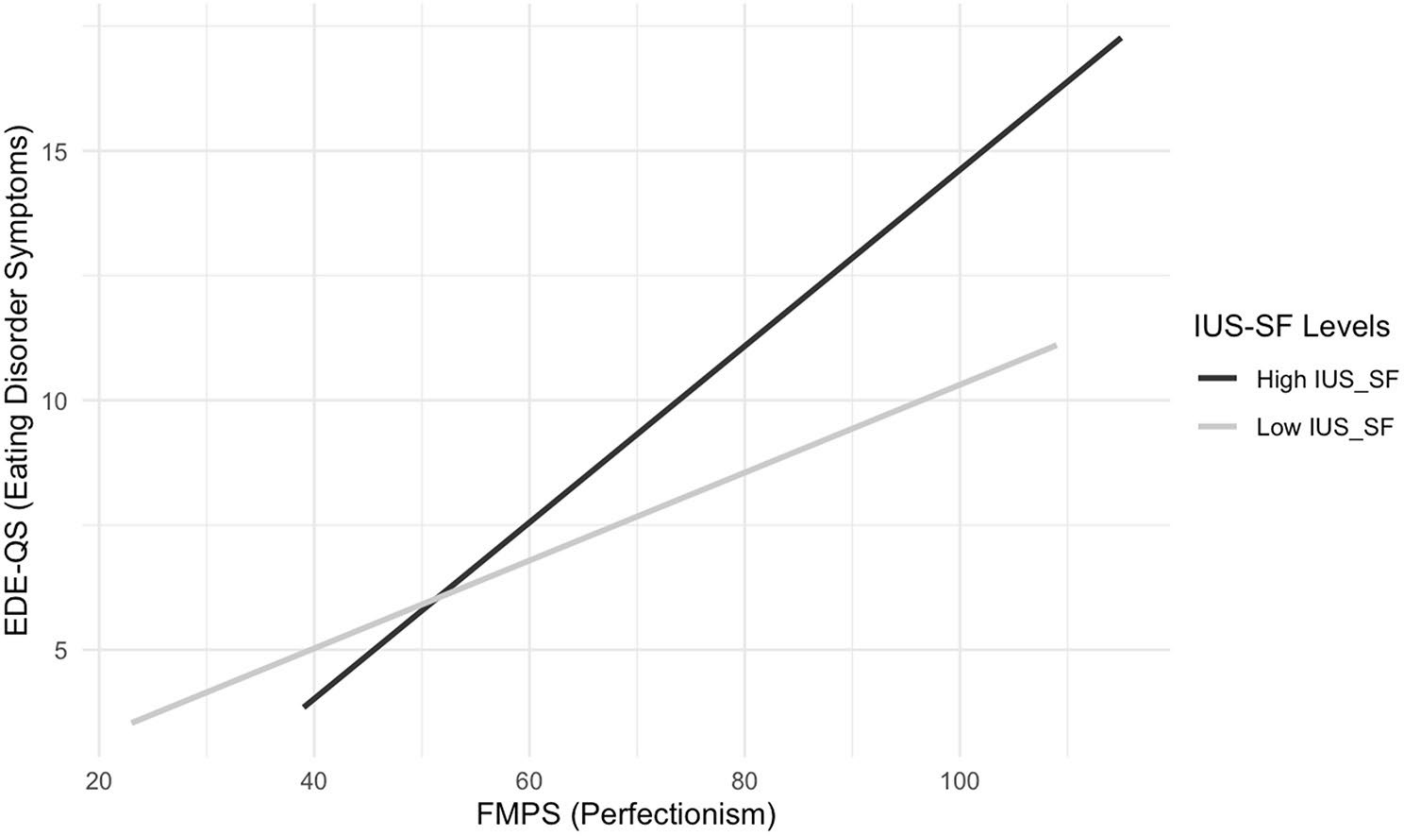
Moderator Model: Moderating Effects Between ED Symptoms, Perfectionism and IU. *Note*: ED (eating disorder), EDE-QS (Eating Disorder Questionnaire Short Form), FMPS (Frost Multidimensional Perfectionism Scale), IUS-SF (Intolerance of Uncertainty Scale). The splits were made using the median value (High IUS-SF > median IUS-SF score; Low IUS-SF < median IUS-SF score)

In the total sample, a multiple linear regression was used to identify if total scores on the FMPS and the interaction between total FMPS scores and total IUS-SF scores significantly predicted EDE-QS scores. This model was statistically significant, *F*(2, 592) = 39.23, *p* < .001, accounting for 11% of the variance of EDE-QS scores. Perfectionism alone was a significant predictor *t*(592) = 2.23, *p* = .026. The interaction between perfectionism and IU was also a significant predictor *t*(592) = 3.37, *p* = .001. These results suggest that both FMPS and the FMPS*IUS-SF interaction are partial moderators of EDE-QS scores (Fig. 1). Standardised coefficients indicate that the effect of perfectionism (0.14) was less significant than the interaction between perfectionism and IU (0.22).

### Interaction between perfectionism, RNT and ED symptoms

**Fig. 2:**
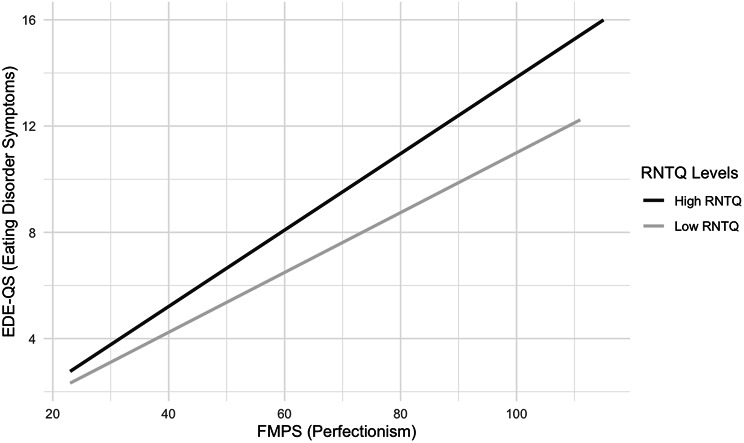
Moderator Model: Moderating Effects Between ED Symptoms, Perfectionism and RNTQ. *Note* ED (eating disorder), EDE-QS (Eating Disorder Questionnaire Short Form), FMPS (Frost Multidimensional Perfectionism Scale), RNTQ (Repetitive Negative Thought Questionnaire. The splits were made using the median value (High RNTQ > median RNTQ score; Low RNTQ < median RNTQ score)

A multiple linear regression was used to identify if total scores on the FMPS and the interaction between total FMPS scores and total RNTQ scores significantly predicted EDE-QS scores. This model was statistically significant, *F*(2, 594) = 45.07, *p* < .001, accounting for 13% of the variance of EDE-QS scores. Perfectionism alone was a non-significant predictor *t*(594) = 1.56, *p* = .126. The interaction between perfectionism and RNT was a significant predictor *t*(592) = 4.65, *p* < .001. These results suggest that both FMPS and the FMPS*RNTQ interaction are partial moderators of EDE-QS scores (Fig. 2). Standardised coefficients suggest that the effect of perfectionism (0.09) was less significant than the interaction between perfectionism and RNT (0.28).

### Predicting clinical symptoms with perfectionism, age and IU interaction

A binary logistic regression classified participants into either clinical or non-clinical ED scores using total FMPS scores, age and the interaction between total FMPS scores and total IUS-SF scores (Table 2). The Odds Ratio (OR) for FMPS was 1.014. This indicates that for each unit increase in FMPS, the odds of the outcome increase by approximately 1.4%. This effect was not statistically significant (likelihood ratio Chi-square = 1.952, *p* = .162). The interaction between FMPS and IUS-SF yielded an OR of 1.0003. This suggests a small positive effect on the odds of the outcome [33], which was statistically significant (likelihood ratio Chi-square = 4.611, *p* = .031). The odds ratio for age was 0.971. This suggests that for each additional year of age, the odds of the outcome decrease by approximately 2.9%. This effect was not statistically significant (likelihood ratio Chi-square = 3.001, *p* = .082).

**Table 2:** Odds ratio, 95% confidence intervals and Wald Chi-Square Test for Binary Logistic Model Predicting clinical ED scores with FMPS, Age and FMPS*IUS-SF

Predictor variable	Odds ratio (OR)	Standard error	95% Confidence interval for OR		LR Chisq	*P* Value
			Lower	Upper		
FMPS	1.014	0.010	0.994	1.034	1.952	0.162
FMPS*IUS-SF	1.0003	< 0.001	1.001	1.001	4.611	0.031
Age	0.971	0.018	0.935	1.004	3.001	0.082

*Note* EDE-QS scores coded as < 15 = 0; > = 15 = 1. FMPS (Frost Multidimensional Perfectionism Scale), IUS-SF (Intolerance of Uncertainty Scale), LR Chisq (Likelihood Ratio Chi-Square)

### Predicting clinical ED symptoms with perfectionism, age and RNT interaction

A similar binary logistic regression was run with the FMPS*RNTQ (Table 3). The OR for FMPS was 1.004. This indicates that for each unit increase in FMPS, the odds of the outcome increase by approximately 0.4%. This effect was not statistically significant (likelihood ratio Chi-square = 0.142, *p* = .706). The interaction between FMPS and RNTQ yielded an OR of 1.0002. This suggests a small positive effect on the odds of the outcome [33], which was statistically significant (likelihood ratio Chi-square = 12.343, *p* < .001). The odds ratio for age was 0.960. This suggests that for each additional year of age, the odds of the outcome decrease by approximately 4%. This effect was statistically significant (likelihood ratio Chi-square = 4.461, *p* = .035).

**Table 3:** Odds ratio, 95% confidence intervals and Wald Chi-square test for Binary Logistic Model Predicting clinical ED scores with FMPS, Age and FMPS*RNTQ

Predictor variable	Odds ratio (OR)	Standard error	95% Confidence interval for OR		LR Chisq	*P* Value
			Lower	Upper		
FMPS	1.004	0.009	0.984	1.023	0.142	0.706
Age	0.960	0.019	0.926	0.997	4.461	0.035
FMPS*RNTQ	1.0002	< 0.001	1.0001	1.0003	12.343	< 0.001

*Note* EDE-QS scores coded as < 15 = 0; > = 15 = 1. FMPS (Frost Multidimensional Perfectionism Scale), RNTQ (Repetitive Negative Thoughts Questionnaire), LR Chisq (Likelihood Ratio Chi-Square)

## Discussion

This study aimed to enhance our understanding of the role that perfectionism, IU and RNT play in EDs by investigating the moderating effect of IU and RNT on the relationship between perfectionism and ED symptoms. Our hypotheses that IU and RNT would significantly moderate this relationship was supported, with results indicating that the interactions had a significant association with ED symptoms. The study also aimed to investigate whether the interaction between perfectionism and IU or RNT could predict clinically significant ED symptoms. Our hypotheses that interactions would predict clinically significant ED symptoms were supported and we found that these interactions had significant predictive power.

### IU and RNT moderate the relationship between perfectionism and ED symptoms

Our findings align with previous studies identifying IU and RNT as partial moderators between perfectionism and psychiatric presentations [15, 22, 25]. However, our study builds on this research by indicating these moderating effects are also specifically relevant to ED symptoms in clinical populations. In addition, our study expands on the findings by Brosof et al. [21] by demonstrating that IU moderates the relationship between total perfectionism and ED symptoms. This indicates that IU interacts with total perfectionism, not only the high-standards subscale as found in Brosof et al. [21]. Similarly, our results also support and expand on the work of Rivière et al. [27] who found that rumination mediated the relationship between ED symptoms and perfectionism. Our findings suggest that other aspects of perfectionism, such as concern over mistakes and worry, specifically contribute to moderating ED symptoms. This would align with a recent study Ralph-Nearman et al. [4] which found that concern over mistakes was strongly linked to ED symptoms. Future studies could benefit from exploring further the relative impact of different perfectionism subscales.

### Moderating effects in a clinical population

Our study found that the IU*perfectionism and RNT*perfectionism interactions predicted clinically significant ED symptoms. This indicates that the moderating effect of IU and RNT has bearing to ED severity in clinical populations. However, it is important to note that these interactions had relatively small predictive power [33]. This could be due to an additive relationship between these constructs, or it may indicate that while significant, the interaction effects are not large. In addition, our study included participants with a range of possible ED diagnoses, suggesting that this moderating effect is relevant across ED diagnoses and supporting the rationale for a transdiagnostic theory of EDs [1, 2]. Understanding this topic further could help with the identification and/or treatment of clinical EDs. For example, Radically Open Dialectical Behaviour Therapy targets perfectionism symptoms in its eating disorder intervention programme [40], whereas cognitive bias modification interpretation retraining aims to reorient biased interpretations [41, 42], which could simultaneously reduce RNT and IU. However, the causal and directional effects of these interactions are still unclear, and future studies using longitudinal designs are warranted.

### Limitations and future directions

There are several limitations which should be considered when interpreting our findings. Firstly, the current study used two separate sub-samples, with statistically significant age differences. In addition, age was found to be a significant predictor in several of the models. This aligns with previous studies that have found age is associated with perfectionism, ED symptoms and RNT [43–45]. Specifically, the studies found that levels of perfectionism, ED symptoms and rumination tended to decrease with age. This is also reflected in some of our findings (Table 3), which demonstrated that each additional year of age was associated with a 4% decrease in the odds of a clinically significant EDE-QS score.

Furthermore, our sample was considerably skewed towards young females, with a mean age of 22.81 and 75% of the sample identifying as female. Although EDs tend to be highly prevalent in young females [46], the composition of our sample limits our ability to generalise our findings. For example, past studies have found gender differences in both RNT and perfectionism [47, 48]. Future studies should aim to feature more diverse and gender-balanced samples. Additionally, regression analysis tends to penalise models with large collinearity and assign larger confidence intervals [13, 49].

Finally, our study used a validated and shortened adaptation of the Eating Disorder Examination Questionnaire (EDQ) to measure ED symptoms. The EDQ has been criticised for having a bias towards the assessment of anorexia nervosa and bulimia nervosa, as well as stereotypically feminine manifestations of EDs [50, 51]. Therefore, future work should include measures that are more robustly applicable to a broader range of EDs and male-identifying individuals. This is important to acknowledge, as BED is estimated to be the most prevalent of all EDs [52] and was the most prevalent ED diagnosis in our sample.

## Conclusion

Our study found that both IU and RNT significantly moderated the relationship between perfectionism and ED symptoms. Furthermore, the interactions were able to discriminate between clinical and non-clinical ED symptoms. Our results advance our understanding of the interactions between perfectionism, IU, RNT and ED symptoms, and may help us better understand, predict, and treat clinical EDs. For example, addressing IU and RNT in addition to perfectionism may yield a greater reduction in ED symptoms. Taken together, the results of the current study support the transdiagnostic theory of EDs by demonstrating that in addition to perfectionism, IU and RNT play a significant role in moderating ED symptomatology across different diagnoses.

## Electronic supplementary material

Below is the link to the electronic supplementary material.

Supplementary Material 1

Supplementary Material 2

Supplementary Material 3

Supplementary Material 4

Supplementary Material 5

Supplementary Material 6

Supplementary Material 7

Supplementary Material 8

Supplementary Material 9

## Data Availability

Data available on request due to privacy/ethical restrictions. The data that support the findings of this study are available on request from the corresponding author. The data are not publicly available due to privacy or ethical restrictions.
