# Supplementary material for: Intolerance of uncertainty and repetitive negative thinking: transdiagnostic moderators of perfectionism in eating disorders
Source: J Eat Disord. 2024 Nov 4;12:173. doi: 10.1186/s40337-024-01138-1 (PMC11536761; doi:10.1186/s40337-024-01138-1)
Supplement: Supplementary file 1 — Supplementary Material 1 [file 40337_2024_1138_MOESM1_ESM.docx]

**S1**

**Highly Prevalent Diagnoses in Clinical Sample Based on Structured Clinical Interview and Associated EDE-QS Scores (*n=*329)**

| **Diagnosis** | **n (% of participants who completed a clinical interview)** | **Mean (SD) EDE-QS Scores** | **% (*n*) of participants with >=15 EDE-QS scores by Diagnoses** |
| --- | --- | --- | --- |
| Major Depressive Disorder | 145 (44%) | 10.89 (8.35) | 30% (43) |
| Persistent Depressive Disorder | 60 (18%) | 9.50 (7.64) | 20% (12) |
| Social Anxiety Disorder | 98 (30%) | 8.87 (7.58) | 18% (18) |
| Panic Disorder | 22 (7%) | 9.57 (4.68) | 14% (3) |
| Generalised Anxiety Disorder | 155 (47%) | 8.42 (6.98) | 17% (26) |
| Obsessive Compulsive Disorder | 22 (7%) | 8.29 (5.95) | 18% (4) |
| Body Dysmorphic Disorder | 53 (16%) | 13.97 (7.58) | 45% (24) |
| Somatic Symptom Disorder | 31 (9%) | 7.95 (8.69) | 23% (7) |
| Insomnia Disorder | 82 (25%) | 7.58 (9.02) | 24% (20) |

*Note:* Individual participants may have received multiple diagnoses. EDE-QS (Eating Disorder Questionnaire Short Form)

**Correlation Co-efficient Between Total EDE-QS Score and Total Number of Comorbidities as per Diagnostic Interview Results (*n=*329)**

| **Correlation Co-efficient** | **p-value** | **95% Confidence Intervals around Co-efficient** | |
| --- | --- | --- | --- |
|  |  | **Lower** | **Upper** |
| 0.114 | .04 | 0.006 | 0.219 |

This result suggests a statistically significant weak positive linear relationship between total number of diagnoses and total scores on the EDE-QS.
