# Supplementary material for: Intolerance of uncertainty and repetitive negative thinking: transdiagnostic moderators of perfectionism in eating disorders
Source: J Eat Disord. 2024 Nov 4;12:173. doi: 10.1186/s40337-024-01138-1 (PMC11536761; doi:10.1186/s40337-024-01138-1)
Supplement: Supplementary file 2 — Supplementary Material 2 [file 40337_2024_1138_MOESM2_ESM.docx]

**S2**

**Participant Flow Chart**

161 participants removed because of incomplete or invalid EDE-QS, FMPS, IUS-SF or RNTQ responses.

Data from 492 participants collected.

**Clinical Sample**

Data from 331 clinical participants included in final analysis.

Note: 329 of these participants had completed diagnostic clinical interviews.

Data from 264 participants collected.

**University Sample**

Total sample size: 595

- 331 participants from Clinical sample
- 264 participants from University sample

**Final Sample**

Clinical participants (*n*=492) were treatment-seeking patients at the University of Melbourne Psychology Clinic (UMPC) who were recruited between 2018 and 2022. The UMPC provides outpatient psychological treatment to the local community, university students and staff. Upon intake, participants completed the questionnaires listed below. In addition, the majority of clinical participants (*n=*390) completed a structured clinical interview.

University participants were 264 undergraduate students from the University of Melbourne who received course credit for completing the same questionnaires as the clinical sample described above. These non-treatment-seeking participants did not complete a structured clinical interview.

Data from 161 clinical participants was excluded because of incomplete and/or invalid responses on the EDE-QS, FMPS, IUS-SF or RNTQ, leaving a total sample of 595 participants (331 clinical participants and 264 undergraduate student participants) for our analyses.
