# Supplementary material for: Intolerance of uncertainty and repetitive negative thinking: transdiagnostic moderators of perfectionism in eating disorders
Source: J Eat Disord. 2024 Nov 4;12:173. doi: 10.1186/s40337-024-01138-1 (PMC11536761; doi:10.1186/s40337-024-01138-1)
Supplement: Supplementary file 3 — Supplementary Material 3 [file 40337_2024_1138_MOESM3_ESM.docx]

**S3**

**Correlation Matrix of EDE-QS, FMPS, RNTQ, IUS-SF and Age for Clinical Sample**

**
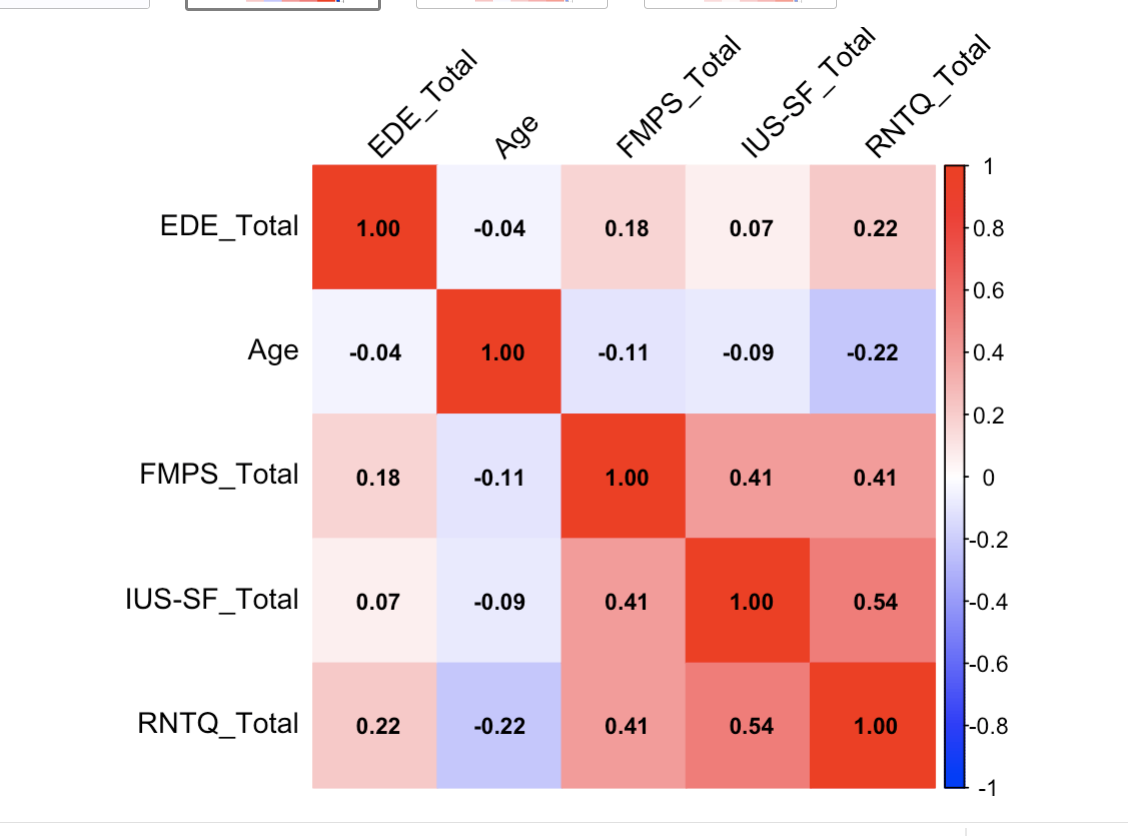
**

*Note:* EDE_Total (Eating Disorder Questionnaire Short Form total score), FMPS_Total (Frost Multidimensional Perfectionism Scale total score), IUS-SF_Total (Intolerance of Uncertainty Scale Short Form total score), RNTQ_Total (Repetitive Negative Thought Questionnaire total score).

**Correlation Matrix of EDE-QS, FMPS, RNTQ, IUS-SF and Age for University Sample**

**
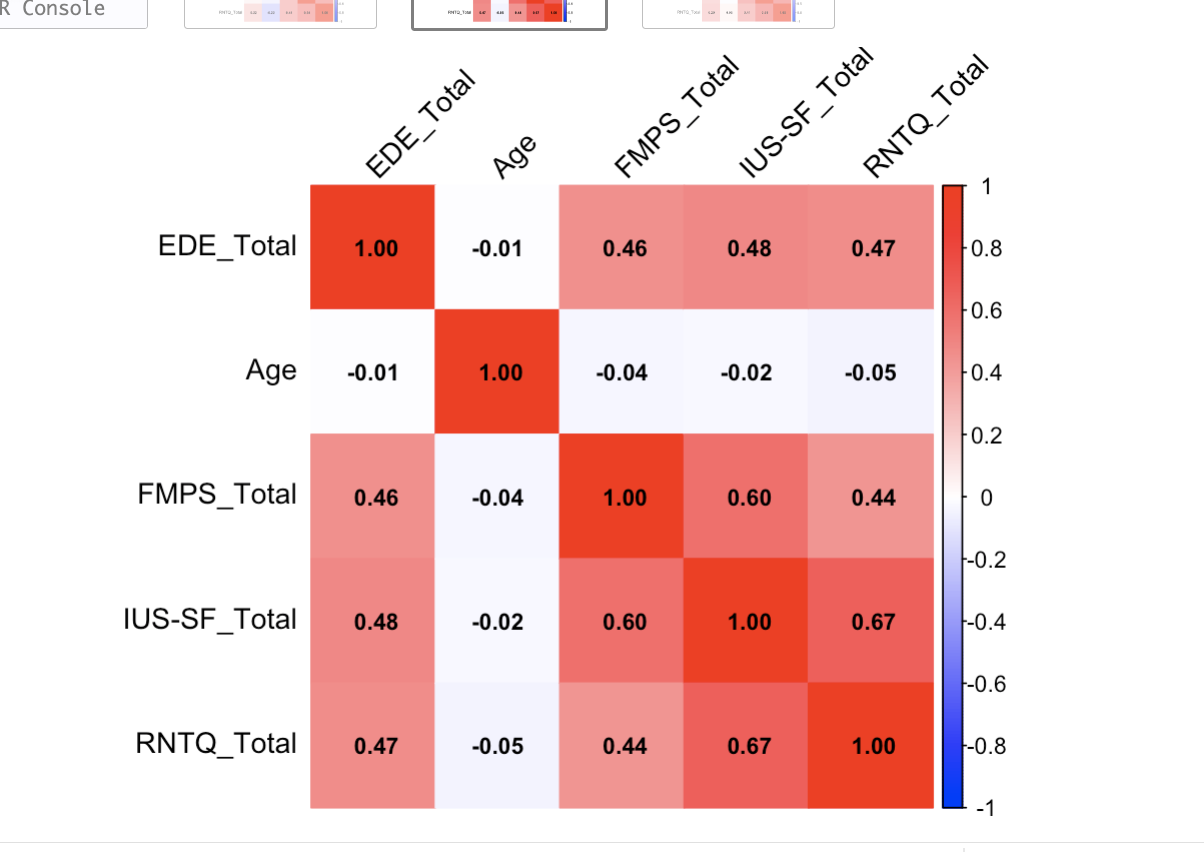
**

*Note:* EDE_Total (Eating Disorder Questionnaire Short Form total score), FMPS_Total (Frost Multidimensional Perfectionism Scale total score), IUS-SF_Total (Intolerance of Uncertainty Scale Short Form total score), RNTQ_Total (Repetitive Negative Thought Questionnaire total score).

**Correlation Matrix of EDE-QS, FMPS, RNTQ, IUS-SF and Age for Total Sample**


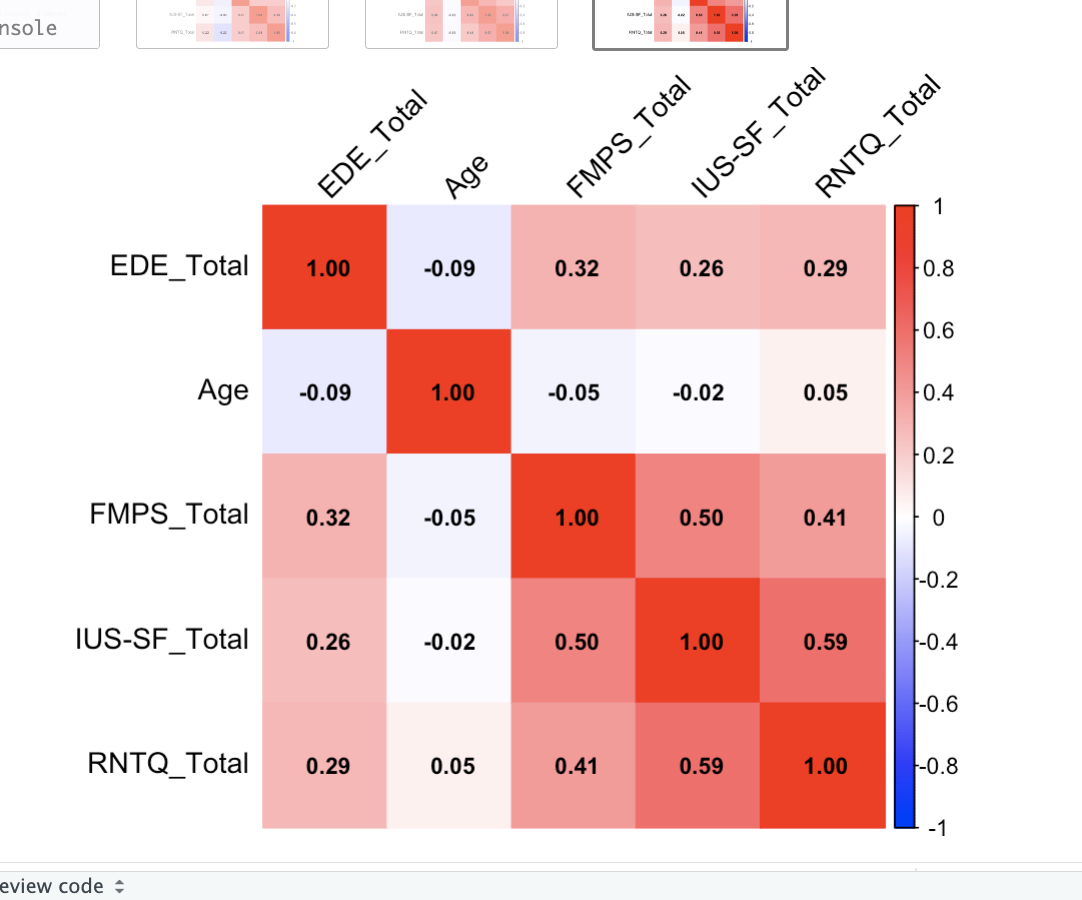


*Note:* EDE_Total (Eating Disorder Questionnaire Short Form total score), FMPS_Total (Frost Multidimensional Perfectionism Scale total score), IUS-SF_Total (Intolerance of Uncertainty Scale Short Form total score), RNTQ_Total (Repetitive Negative Thought Questionnaire total score).
