# Supplementary material for: Intolerance of uncertainty and repetitive negative thinking: transdiagnostic moderators of perfectionism in eating disorders
Source: J Eat Disord. 2024 Nov 4;12:173. doi: 10.1186/s40337-024-01138-1 (PMC11536761; doi:10.1186/s40337-024-01138-1)
Supplement: Supplementary file 4 — Supplementary Material 4 [file 40337_2024_1138_MOESM4_ESM.docx]

**S3**

**Examining Influence of Age on Models**

*Age as Covariate Model 1 using Standardised Estimates (Outcome: EDE-QS, Predictors: FMPS, IUS-SF, RNTQ, Age).*

|  | Estimate | Std. Estimate | Std. Error | t value | p-value |
| --- | --- | --- | --- | --- | --- |
| Intercept | -1.57 | NA | 1.82 | -0.86 | .40 |
| FMPS | 0.10 | 0.22 | 0.02 | 4.85 | <.001 |
| Age | -0.1 | -0.08 | 0.05 | -2.22 | .03 |
| IUS-SF | 0.04 | 0.05 | 0.04 | 0.97 | 0.33 |
| RNTQ | 0.05 | 0.17 | 0.01 | 3.60 | <.001 |
| Residual Standard error | 7.52 on 592 degrees of freedom | | | | |
| Multiple R-squared | 0.14 | | | | |
| Adjusted R-squared | 0.13 | | | | |
| F-statistic | 23.76 on 4 and 592 DF, p-value: < .001 | | | | |

*Note:* EDE-QS (Eating Disorder Questionnaire Short Form), FMPS (Frost Multidimensional Perfectionism Scale), IUS-SF (Intolerance of Uncertainty Scale Short Form), RNTQ (Repetitive Negative Thinking Questionnaire).

*Age as Covariate Model 2 using Standardised Estimates (Outcome: EDE-QS, Predictors: FMPS, IUS-SF, Age).*

|  | Estimate | Std. Estimate | Std. Error | t value | p-value |
| --- | --- | --- | --- | --- | --- |
| Intercept | -0.90 | NA | 1.83 | -0.49 | .62 |
| FMPS | 0.12 | 0.24 | 0.02 | 5.46 | <.001 |
| Age | -0.09 | -0.07 | 0.05 | -1.90 | .06 |
| IUS-SF | 0.10 | 0.14 | 0.03 | 3.11 | <.001 |
| Residual Standard error | 7.6 on 593 degrees of freedom | | | | |
| Multiple R-squared | 0.12 | | | | |
| Adjusted R-squared | 0.11 | | | | |
| F-statistic | 26.83 on 3 and 593 DF, p-value: < .001 | | | | |

*Note:* EDE-QS (Eating Disorder Questionnaire Short Form), FMPS (Frost Multidimensional Perfectionism Scale), IUS-SF (Intolerance of Uncertainty Scale Short Form).

*Age as Covariate Model 3 using Standardised Estimates (Outcome: EDE-QS, Predictors: FMPS, RNTQ, Age).*

|  | Estimate | Std. Estimate | Std. Error | t value | p-value |
| --- | --- | --- | --- | --- | --- |
| Intercept | -1.35 | NA | 1.81 | -0.75 | .45 |
| FMPS | 0.11 | 0.23 | 0.02 | 5.53 | <.001 |
| Age | -0.10 | -0.09 | 0.05 | -2.25 | .02 |
| RNTQ | 0.06 | 0.20 | 0.01 | 4.67 | <.001 |
| Residual Standard error | 7.52 on 593 degrees of freedom | | | | |
| Multiple R-squared | 0.14 | | | | |
| Adjusted R-squared | 0.13 | | | | |
| F-statistic | 31.37 on 3 and 593 DF, p-value: < .001 | | | | |

*Note:* EDE-QS (Eating Disorder Questionnaire Short Form), FMPS (Frost Multidimensional Perfectionism Scale), RNTQ (Repetitive Negative Thinking Questionnaire).

*Age as Covariate Model 4 using Standardised Estimates (Outcome: EDE-QS, Predictors: FMPS, FMPS*IUS-SF, Age).*

|  | Estimate | Std. Estimate | Std. Error | t value | p-value |
| --- | --- | --- | --- | --- | --- |
| Intercept | 2.50 | NA | 1.92 | 1.30 | .19 |
| FMPS_Total | 0.07 | 0.14 | 0.03 | 2.18 | .03 |
| Age | -0.09 | -0.07 | 0.05 | -1.86 | .06 |
| FMPS*IUS-SF | 0.001 | 0.22 | 0.004 | 3.34 | <.001 |
| Residual Standard error | 7.58 on 593 degrees of freedom | | | | |
| Multiple R-squared | 0.12 | | | | |
| Adjusted R-squared | 0.12 | | | | |
| F-statistic | 27.47 on 3 and 593 DF, p-value: < .001 | | | | |

*Note:* EDE-QS (Eating Disorder Questionnaire Short Form), FMPS (Frost Multidimensional Perfectionism Scale), IUS-SF (Intolerance of Uncertainty Scale Short Form).

*Age as Covariate Model 5 using Standardised Estimates (Outcome: EDE-QS, Predictors: FMPS, FMPS*RNTQ, Age)*

|  | Estimate | Std. Estimates | Std. Error | t value | p-value |
| --- | --- | --- | --- | --- | --- |
| Intercept | 3.58 | NA | 1.91 | 1.87 | .06 |
| FMPS_Total | 0.04 | 0.08 | 0.03 | 1.34 | .19 |
| Age | -0.11 | -0.09 | 0.05 | -2.28 | .02 |
| FMPS*RNTQ | 0.001 | 0.29 | 0.0002 | 4.85 | <.001 |
| Residual Standard error | 7.51 on 593 degrees of freedom | | | | |
| Multiple R-squared | 0.14 | | | | |
| Adjusted R-squared | 0.13 | | | | |
| F-statistic | 32 on 3 and 593 DF, p-value: <.001 | | | | |

*Note:* EDE-QS (Eating Disorder Questionnaire Short Form), FMPS (Frost Multidimensional Perfectionism Scale), RNTQ (Repetitive Negative Thinking Questionnaire).
