# Supplementary material for: Intolerance of uncertainty and repetitive negative thinking: transdiagnostic moderators of perfectionism in eating disorders
Source: J Eat Disord. 2024 Nov 4;12:173. doi: 10.1186/s40337-024-01138-1 (PMC11536761; doi:10.1186/s40337-024-01138-1)
Supplement: Supplementary file 5 — Supplementary Material 5 [file 40337_2024_1138_MOESM5_ESM.docx]

**S4**

**Normality Assumption Check Results**

Shapiro-Wilk normality test EDE-QS entire sample
W = 0.905, p-value < .001

Shapiro-Wilk normality test IUS-SF entire sample
W = 0.989, p-value < .001

Shapiro-Wilk normality test RNTQ entire sample
W = 0.965, p-value < .001

Shapiro-Wilk normality test FMPS entire sample
W = 0.994, p-value = .031

*
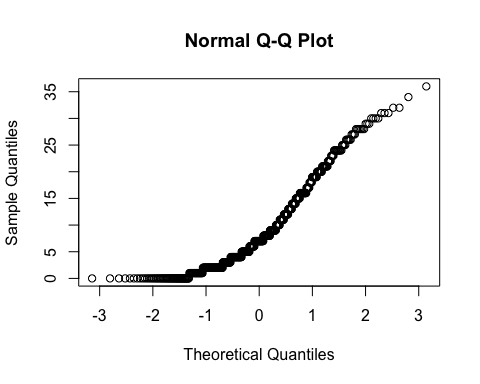
*

*EDE-QS Q-Q Plot Entire Sample*


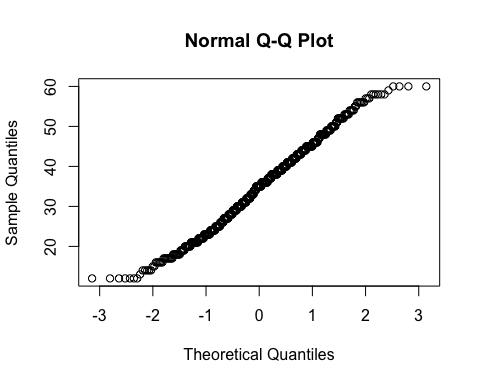


*IUS-SF Q-Q Plot Entire Sample*


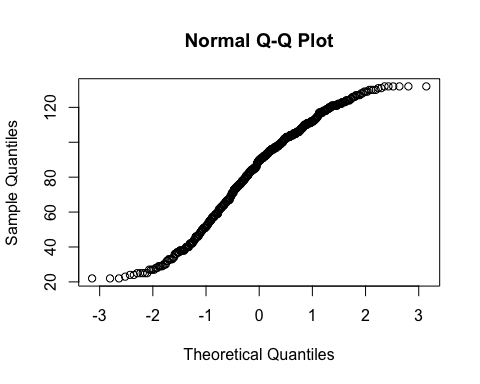


*RNTQ Q-Q Plot Entire Sample*


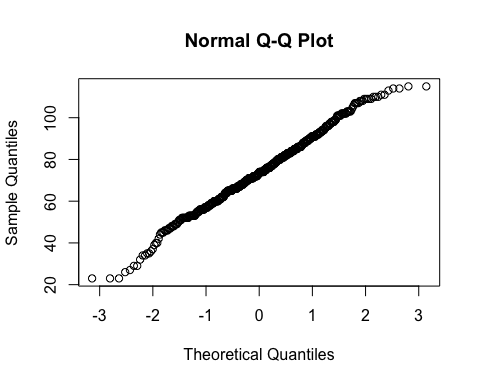


*FMPS Q-Q Plot Entire Sample*

Checking normality of standardised residuals of linear regression model 1
Shapiro-Wilk normality test
W = 0.940, p-value < .001

Checking normality of standardised residuals of linear regression model 2

Shapiro-Wilk normality test
W = 0.949, p-value < .001
