# Supplementary material for: Intolerance of uncertainty and repetitive negative thinking: transdiagnostic moderators of perfectionism in eating disorders
Source: J Eat Disord. 2024 Nov 4;12:173. doi: 10.1186/s40337-024-01138-1 (PMC11536761; doi:10.1186/s40337-024-01138-1)
Supplement: Supplementary file 6 — Supplementary Material 6 [file 40337_2024_1138_MOESM6_ESM.docx]

**S5**

**Linearity Assumption Check Results**


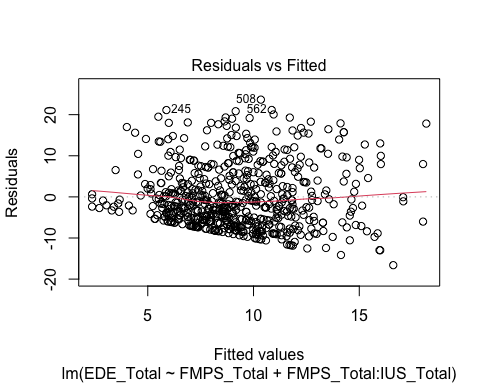


*Residuals vs Fitted Values for Linear Regression Model: Total FMPS and total FMPS/total IUS-SF interaction*


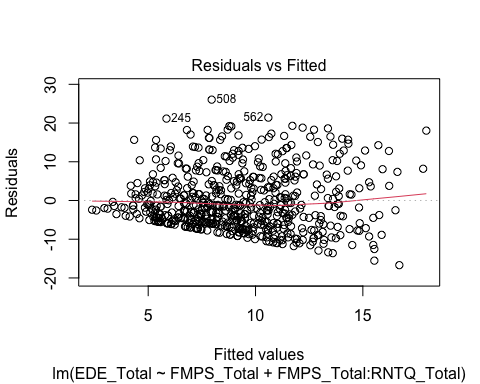


*Residuals vs Fitted Values for Linear Regression Model: Total FMPS and total FMPS/total RNTQ interaction*

*Checking Linearity for Each Predictor* *for Linear Regression Model: Total FMPS and total FMPS/total IUS-SF interaction*


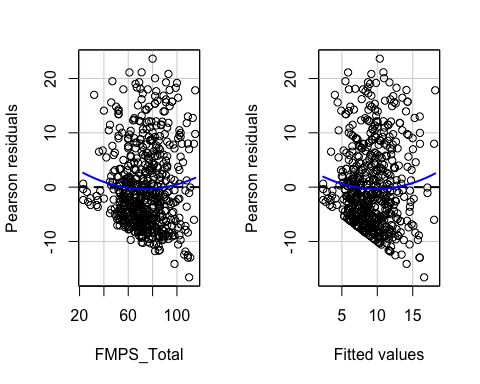


Test stat Pr(>|Test stat|)
FMPS_Total 1.564 0.118
Tukey test 1.496 0.134

*Checking Linearity for Each Predictor* *for Linear Regression Model: Total FMPS and total FMPS/total RTNQ interaction*


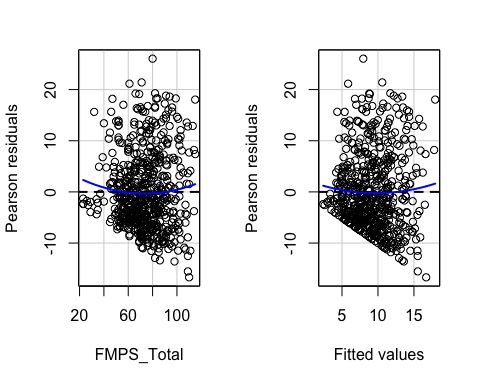


Test stat Pr(>|Test stat|)
FMPS_Total 1.396 0.163

Tukey test 0.937 0.348
