# Supplementary material for: Intolerance of uncertainty and repetitive negative thinking: transdiagnostic moderators of perfectionism in eating disorders
Source: J Eat Disord. 2024 Nov 4;12:173. doi: 10.1186/s40337-024-01138-1 (PMC11536761; doi:10.1186/s40337-024-01138-1)
Supplement: Supplementary file 7 — Supplementary Material 7 [file 40337_2024_1138_MOESM7_ESM.docx]

**S6**

**Collinearity Assumption Checks**

Typically, variation inflation factors larger than 2 or 3 indicate possible problems with collinearity. Therefore, our results appear to be within this boundary.

*Variance Inflation Factor Values for Measures and Models*

| **Measures and Model** | **Variance Inflation Factor** |
| --- | --- |
| Linear Regression Model:  Total FMPS and total FMPS/total IUS-SF interaction | 2.77 |
| Linear Regression Model:  Total FMPS and total FMPS/total RNTQ interaction | 2.54 |
| Logistic Regression Model:  Total FMPS and total FMPS/total IUS-SF interaction | 2.64 |
| Logistic Regression Model:  Total FMPS and total FMPS/total RNTQ interaction | 2.63 |

FMPS (Frost Multidimensional Perfectionism Scale), RNTQ (Repetitive Negative Thoughts Questionnaire), IUS-SF (Intolerance of Uncertainty Scale Short Form).
