# Supplementary material for: Intolerance of uncertainty and repetitive negative thinking: transdiagnostic moderators of perfectionism in eating disorders
Source: J Eat Disord. 2024 Nov 4;12:173. doi: 10.1186/s40337-024-01138-1 (PMC11536761; doi:10.1186/s40337-024-01138-1)
Supplement: Supplementary file 8 — Supplementary Material 8 [file 40337_2024_1138_MOESM8_ESM.docx]

**S7**

**AIC and BIC Results, Model Selection**

*AIC and BIC results for model 1 multiple linear regression models (bolded = selected model)*

| **Model Description** | **AIC** | **BIC** |
| --- | --- | --- |
| Outcome: EDE-QS Scores  Predictors: FMPS, IUS-SF and FMPS:IUS-SF interaction | 4122.39 | 4144.35 |
| **Outcome: EDE-QS Scores**  **Predictors: FMPS and FMPS*IUS-SF interaction** | 4120.61 | 4138.18 |

EDE_Total (Eating Disorder Questionnaire Short Form total score), FMPS_Total (Frost Multidimensional Perfectionism Scale total score), IUS-SF_Total (Intolerance of Uncertainty Scale Short Form total score), RNTQ_Total (Repetitive Negative Thought Questionnaire total score).

*AIC and BIC results for model 2 multiple linear regression models (bolded = selected model)*

| **Model Description** | **AIC** | **BIC** |
| --- | --- | --- |
| Outcome: EDE-QS Scores  Predictors: FMPS, RNTQ and FMPS:RNTQ interaction | 4112.481 | 4134.44 |
| **Outcome: EDE-QS Scores**  **Predictors: FMPS and FMPS*RNTQ interaction** | 4110.48 | 4128.05 |

EDE_Total (Eating Disorder Questionnaire Short Form total score), FMPS_Total (Frost Multidimensional Perfectionism Scale total score), IUS-SF_Total (Intolerance of Uncertainty Scale Short Form total score), RNTQ_Total (Repetitive Negative Thought Questionnaire total score).
