# Supplementary material for: Intolerance of uncertainty and repetitive negative thinking: transdiagnostic moderators of perfectionism in eating disorders
Source: J Eat Disord. 2024 Nov 4;12:173. doi: 10.1186/s40337-024-01138-1 (PMC11536761; doi:10.1186/s40337-024-01138-1)
Supplement: Supplementary file 9 — Supplementary Material 9 [file 40337_2024_1138_MOESM9_ESM.docx]

**S9**

**Odds Ratios (OR), 95% Confidence Intervals for OR and Wald Chi-square test for Binary Logistic Regression Models**

*Outcome Variable: EDE-QS scores of =>15. Predictor Variables: Age, Total FMPS, IUS-SF, RNTQ*

| **Variable** | **Odds Ratio (OR)** | **Standard Error** | **Lower CI** | **Upper CI** | **LR Chisq** | **P Value** |
| --- | --- | --- | --- | --- | --- | --- |
| FMPS | 1.019 | 0.007 | 1.006 | 1.033 | 7.834 | .005 |
| IUS-SF | 1.007 | 0.012 | 0.984 | 1.031 | 0.377 | .539 |
| RNTQ | 1.014 | 0.005 | 1.005 | 1.024 | 9.169 | .002 |
| Age | 0.963 | 0.019 | 0.925 | 0.997 | 4.517 | .033 |

*Note:* EDE-QS scores coded as <15=0; >=15=1. EDE-QS (Eating Disorder Questionnaire Short Form), FMPS (Frost Multidimensional Perfectionism Scale), IUS-SF (Intolerance of Uncertainty Scale), RNTQ (Repetitive Negative Thoughts Questionnaire), LR Chisq (Likelihood Ratio Chi-Square), Confidence Intervals (CI) are 95% CI for OR.

*Outcome Variable: EDE-QS scores of =>15. Predictor Variables: Age, Total FMPS, IUS-SF*

| **Variable** | **Odds Ratio (OR)** | **Standard Error** | **Lower CI** | **Upper CI** | **LR Chisq** | **P Value** |
| --- | --- | --- | --- | --- | --- | --- |
| FMPS | 1.023 | 0.007 | 1.009 | 1.037 | 11.322 | .001 |
| IUS-SF | 1.025 | 0.010 | 1.004 | 1.046 | 5.496 | .019 |
| Age | 0.970 | 0.018 | 0.935 | 1.003 | 3.135 | .076 |

*Note:* EDE-QS scores coded as <15=0; >=15=1. EDE-QS (Eating Disorder Questionnaire Short Form), FMPS (Frost Multidimensional Perfectionism Scale), IUS-SF (Intolerance of Uncertainty Scale), LR Chisq (Likelihood Ratio Chi-Square), Confidence Intervals (CI) are 95% CI for OR.

*Outcome Variable: EDE-QS scores of =>15. Predictor Variables: Age, Total FMPS, RNTQ*

| **Variable** | **Odds Ratio (OR)** | **Standard Error** | **Lower CI** | **Upper CI** | **LR Chisq** | **P Value** |
| --- | --- | --- | --- | --- | --- | --- |
| FMPS | 1.021 | 0.006 | 1.008 | 1.034 | 10.401 | .001 |
| RNTQ | 1.016 | 0.004 | 1.007 | 1.024 | 14.288 | <.001 |
| Age | 0.962 | 0.019 | 0.925 | 0.997 | 4.611 | .031 |

*Note:* EDE-QS scores coded as <15=0; >=15=1. EDE-QS (Eating Disorder Questionnaire Short Form), FMPS (Frost Multidimensional Perfectionism Scale), IRNTQ (Repetitive Negative Thoughts Questionnaire), LR Chisq (Likelihood Ratio Chi-Square), Confidence Intervals (CI) are 95% CI for OR.

*Outcome Variable: EDE-QS scores of =>15. Predictor Variables: Age, Total FMPS, FMPS*IUS-SF*

| **Variable** | **Odds Ratio (OR)** | **Standard Error** | **Lower CI** | **Upper CI** | **LR Chisq** | **P Value** |
| --- | --- | --- | --- | --- | --- | --- |
| FMPS | 1.014 | 0.010 | 0.994 | 1.034 | 1.952 | .162 |
| FMPS*IUS-SF | 1.0003 | <0.001 | 1.001 | 1.001 | 4.611 | .031 |
| Age | 0.971 | 0.018 | 0.935 | 1.004 | 3.001 | .082 |

*Note:* EDE-QS scores coded as <15=0; >=15=1. EDE-QS (Eating Disorder Questionnaire Short Form), FMPS (Frost Multidimensional Perfectionism Scale), IUS-SF (Intolerance of Uncertainty Scale), LR Chisq (Likelihood Ratio Chi-Square), Confidence Intervals (CI) are 95% CI for OR.

*Outcome Variable: EDE-QS scores of =>15. Predictor Variables: Age, Total FMPS, FMPS*RNTQ*

| **Variable** | **Odds Ratio (OR)** | **Standard Error** | **Lower CI** | **Upper CI** | **LR Chisq** | **P Value** |
| --- | --- | --- | --- | --- | --- | --- |
| FMPS | 1.004 | 0.010 | 0.984 | 1.023 | 0.142 | .706 |
| FMPS*RNTQ | 1.0002 | <0.001 | 1.000 | 1.001 | 12.343 | <.001 |
| Age | 0.963 | 0.019 | 0.926 | 0.997 | 4.461 | .035 |

*Note:* EDE-QS scores coded as <15=0; >=15=1. EDE-QS (Eating Disorder Questionnaire Short Form), FMPS (Frost Multidimensional Perfectionism Scale), RNTQ (Repetitive Negative Thoughts Questionnaire), LR Chisq (Likelihood Ratio Chi-Square), Confidence Intervals (CI) are 95% CI for OR.
